# In Vitro Antibacterial Activity of Essential Oils from *Origanum vulgare*, *Satureja montana*, *Thymus vulgaris*, and Their Blend Against Necrotoxigenic (NTEC), Enteropathogenic (EPEC), and Shiga-Toxin Producing *Escherichia coli* (STEC) Isolates

**DOI:** 10.3390/pathogens13121077

**Published:** 2024-12-08

**Authors:** Giulia Cagnoli, Fabrizio Bertelloni, Valentina Virginia Ebani

**Affiliations:** 1 Department of Veterinary Sciences, University of Pisa, 56124 Pisa, Italy; giulia.cagnoli@phd.unipi.it (G.C.); fabrizio.bertelloni@unipi.it (F.B.); 2 Interdepartmental Research Center “Nutraceuticals and Food for Health” (NUTRAFOOD), University of Pisa, 56121 Pisa, Italy

**Keywords:** essential oils, antibacterial activity, *Escherichia coli* pathotypes, fecal contamination

## Abstract

Enteropathogenic (EPEC), necrotoxigenic (NTEC), and Shiga-toxin producing *Escherichia coli* (STEC) are pathotypes responsible for severe clinical forms in humans and animals. They can be shed in the feces of animals with consequent environmental contamination. This study evaluated the antibacterial activity of essential oils (EOs) from oregano (*Origanum vulgare)*, savory *(Satureja montana)*, thyme (*Thymus vulgaris)*, and their blend against EPEC, NTEC, and STEC strains previously isolated from avian fecal samples. Minimum inhibitory concentration values between 0.039% and 0.156% were found with *O. vulgare* EO, between ≤0.0195% and 0.156% with both *S. montana* and *T. vulgaris* EOs, and between 0.039% and ≤0.0195% with the blend. The mixture with equal parts of EOs from oregano, savory and thyme seems a promising alternative product to combat pathogenic *E. coli* strains responsible for environmental contamination.

## 1. Introduction

*Escherichia coli* is a Gram-negative, non-sporulating, rod-shaped, facultative anaerobic, and coliform bacterium belonging to the family *Enterobacteriaceae*, order *Enterobacterales* [1]. It is known to be an opportunistic agent commonly present in the intestinal tract of animals and humans. It can live for long periods in feces, soil, and water, and is frequently used as a water contamination indicator organism [1]. However, this bacterial species includes some pathotypes which can cause intestinal and extra-intestinal infections. Urinary and genital tract infections, meningitis, and septicemia are the most common extra-intestinal forms both in animals and humans [2]. Intestinal infections are related to *E. coli* diarrhoeagenic pathotypes that act with different mechanisms in relation to their virulence characters. Among them, enteropathogenic *E. coli* (EPEC) causes the loss of intestinal microvillus and induces a high child mortality rate mainly in developing countries [3]. Shiga-toxin producing *E. coli* (STEC) strains are responsible for potentially life-threatening diarrhea with complications such as hemorrhagic colitis (HC) and diarrhea-associated hemolytic-uremic syndrome (HUS) [4]. Necrotoxigenic *E. coli* (NTEC) strains were reported for the first time in neonatal enteritis [5]; in humans, they are associated with dysenteric syndrome, but they are also frequently associated with extra-intestinal infections, such as those of the urinary tract [6]. Strains belonging to all these pathotypes are severe threats for human health, but companion and livestock animals may be affected as well [1,7,8,9]. Moreover, animals, even if asymptomatic, may harbor the pathotypes, becoming sources of infections for humans and other animals [10]. In particular, feces of infected animals, including wild birds [11,12,13,14,15,16], may be responsible for environmental contamination, not only of large areas (farm, pastures, parks) but also small areas (cages, house rooms and balconies) and instruments that could act as fomites. *Escherichia coli* dissemination is a also severe threat in human and veterinary medicine because *E. coli* strains are frequently resistant to many antimicrobials. Several studies, carried out worldwide, detected *E. coli* strains, including diarrhoeagenic ones, resistant to beta-lactams, quinolones, fluoroquinolones, aminoglycosides, fosfomycin, tetracyclines, phenicols, sulphonamides, trimethoprim, and polymyxins [15,17,18].

Essential oils (EOs) are secondary metabolites extracted from leaves, stems, flowers, seeds, roots, fruit rinds, resins, or barks by distillation or mechanical processes [19]. EOs have been known since ancient times and used in perfumery as well as in folk medicine for their antioxidant, anti-inflammatory, antiviral, antimycotic, and antiparasitic properties [19,20,21,22]. Even though their antibacterial activity is known, the study of this property is of increasing interest because therapies with alternative drugs are welcome to combat infections caused by antibiotic-resistant strains, which represent a growing and severe threat in human and veterinary medicine. EOs have also been suggested as promising alternative for environmental applications to control undesired agents [23]. These studies are necessary because the EOs’ antibacterial activity is highly variable in relations to several factors concerning both EOs, such as botanical species and chemical composition, and bacteria. In particular, differences can be observed testing the same EO with bacteria of different species and among strains belonging to a given species [22]. EOs extracted from different botanical species, such as *Mentha piperita*, *Ocimum basilicum*, *Cymbopogon citratus*, *Origanum vulgare*, *Thymus vulgaris*, and *Satureja montana*, have been proven to be active against *E. coli* [22], although differences may be detected in relation to the analyzed *E. coli* strains [22]. No data about EOs’ activity against NTEC and EPEC are available, and some information about STEC regards only *E.coli* strains of food origin [24].

The aim of the present study was to verify the potential in vitro antimicrobial activity of three commercial EOs, obtained from oregano (*Origanum vulgare* L. subsp. *hirticum*), savory (*Satureja montana* L.), and thyme (*Thymus vulgaris* L.), and their blend against previously isolated EPEC, STEC, and NTEC strains.

## 2. Materials and Methods

### 2.1. Essential Oils

Three commercial EOs (FLORA^®^, Pisa, Italy), extracted from oregano (*Origanum vulgare* L. subsp. *hirticum*), savory (*Satureja montana* L.), and thyme (*Thymus vulgaris* L.), were selected for the survey. A blend, containing equal parts of the three oils, was included in the investigation. EOs and blend were stored at 4 °C in dark glass vials and submitted to microbiological analysis for quality control. In addition, the three EOs and their mixture were submitted to Gas Chromatography—Mass Spectrometry Analysis, as previously reported [25].

### 2.2. Antibacterial Activity

#### 2.2.1. Bacterial Strains

Ten *E. coli* isolates were employed in the study. The strains were previously cultured from fecal samples of wild birds. After isolation and identification of the bacterial species, the strains were submitted to molecular analyses to detect virulence genes reportable to different *E. coli* pathotypes [26,27,28]. In details, 5 NTEC, 4 EPEC, all isolated from seagulls (*Larus michahellis*), and 1 STEC, cultured from an owl (*Athene noctua*), were employed in the survey. The isolates were stored at −80 °C in Brain–Heart Infusion (BHI) broth (Oxoid Ltd., Basingstoke, Hampshire, UK), with the addition of 30% glycerol. Before the in vitro antimicrobial activity analyses, the isolates were cultured in BHI broth at 37 °C for 24 h. Cultures of 1–2 × 10^7^ CFU/mL, corresponding to 0.5 McFarland standard, were employed in the analyses.

#### 2.2.2. Antimicrobial Sensitivity Test

All *E. coli* isolates were submitted to the Kirby–Bauer disk diffusion test, following CLSI guidelines [29], to evaluate their resistance to 15 antibiotic molecules. The following antimicrobial disks (Oxoid, Ltd.) were used: ampicillin (10 µg), amoxicillin-clavulanate (20/10 µg), cefoxitin (30 µg), cefotaxime (30 µg), ceftiofur (30 µg), imipenem (10 µg), ertapenem (10 µg), aztreonam (30 µg), chloramphenicol (30 µg), tetracycline (30 µg), enrofloxacin (5 µg), ciprofloxacin (5 µg), gentamicin (10 µg), amikacin (30 µg), and trimethoprim-sulfamethoxazole (1.25–23.75 µg). The obtained growth inhibition zones were interpretated following CLSI [30] criteria.

#### 2.2.3. Essential Oils Minimum Inhibitory Concentration

The sensitivity of the bacterial strains to the three EOs and their mixture was evaluated with the determination of the minimum inhibitory concentration (MIC). For this purpose, the broth microdilution method was executed for each isolate, following the guidelines reported by CLSI [31] and a protocol previously described [32]. The assay was carried out in BHI broth + 1% dimethyl sulfoxide (DMSO, Oxoid). The MIC value was the lowest concentration, expressed in the percentage (10%, 5%, 2.5%, 1.25%, 0.625%, 0.312%, 0.156%, 0.078%, 0.039%, 0.0195% (*v*/*v*)) of each EO and mixture at which bacteria show no visible growth. The test was executed at the same time to check bacterial growth (tested strain and medium) and sterility (tested EO and medium). All tests were carried out in triplicate.

## 3. Results

The disk diffusion test found that two NTEC and the STEC isolates were resistant to amoxicillin-clavulanate and ampicillin, and one NTEC strain was resistant to trimethoprim-sulfamethoxazole. In detail, the strains NTEC 4, NTEC 5, and STEC had growth inhibition zones of 11 mm, 10 mm, and 11 mm, respectively, with amoxicillin-clavulanate, whereas the diameters were of 12 mm, 10 mm, and 11 mm, respectively, with ampicillin. The strain NTEC 2 had an inhibition zone of 8 mm for trimethoprim-sulfamethoxazole. In fact, CLSI defined an *Enterobacteriaceae* strain as sensitive when the growth inhibition zone is ≥16 mm, intermediate 15–11 mm, and resistant ≤ 10 mm for trimethoprim-sulfamethoxazole; sensitive ≥ 18 mm, intermediate 17–14 mm, and resistant ≤ 13 mm for amoxicillin-clavulanate; sensitive ≥ 17 mm, intermediate 16–14 mm, and resistant ≤ 13 mm for ampicillin [29].

The three EOs and their blend showed activity against all ten analyzed *E. coli* isolates. In detail, MIC values ranging from 0.039% to 0.156% were found with *O. vulgare* EO and from ≤0.0195% to 0.156% with both *S. montana* and *T. vulgaris* EOs; MIC values obtained by testing the strains with the mixture were ≤0.0195% for seven isolates (three NTEC and four EPEC strains) and 0.039% for the remaining three isolates (one STEC and two EPEC strains). The results are summarized in Table 1.

## 4. Discussion

Even though most *E. coli* strains are commensal members of the intestinal microbiota of animals and humans, some strains, belonging to pathotypes such as EPEC, STEC, and NTEC, are important pathogens that compromise the health of animals and humans. To the best of our knowledge, the present study is the first that has investigated the activity of oregano, thyme, and savory EOs against field strains of *E. coli* belonging to EPEC, STEC, and NTEC pathotypes. In fact, usually the studies are focused on *E. coli* strains not belonging to particular pathotypes; some investigations described the antimicrobial activity of *T. vulgaris* and *O. vulgare* against *E. coli* cultured from animals and humans [25,33,34,35], and the effectiveness of *S. montana* EO against avian *E. coli*, in particular strains isolated from poultry, was demonstrated [35,36]. On the other hand, data regarding specific *E. coli* pathotypes come from studies on the activity of different EOs only against *E. coli* O157:H7, a STEC serotype, in food products [24,37,38,39].

The analyses of the present study showed promising results; in fact, all EOs had good activity against all analyzed *E. coli* strains, and enhanced antibacterial properties were found using the mixture. The chemical compositions of the EOs and the blend used in the present investigation were previously reported [25]. Carvacrol and thymol comprised the main components in EOs from *O. vulgare* and *T. vulgaris*, respectively; moreover, both carvacrol and thymol were the major compounds in the *S. montana* EO. In details, carvacrol constituted 78.0%, 60.0%, 5.5%, and 50.8% of the whole composition of EOs from oregano, savory, thyme, and of the blend, respectively. Percentages of thymol of 43.6%, 9.5%, 6.9%, and 17.1% were found in thyme, oregano, and savory EOs, and the blend, respectively [25]. These compounds are known to be active against bacteria, destroying their cell wall and cytoplasmic membrane and consequently causing cell lysis and leakage of intracellular compounds [40,41,42].

EOs and the mixture tested in the present study were also previously employed to evaluate their activity against *E. coli* strains isolated from the ears of dogs with otitis. Both the EOs and the blend showed activity to the clinical isolates, with MIC values between 0.078% and 1.25% for oregano, 0.078% and 0.156% for savory, 0.156% and 0.625% (one strain >10%) for thyme, and ≤0.0195% and 0.078% for the mixture [25]. The results obtained in the present investigation confirmed the antimicrobial activity of these EOs against *E. coli*, but even NTEC, EPEC, and STEC strains seem to be more susceptible, mainly when tested with the blend.

The *E. coli* strains assayed in the present investigation belong to pathotypes particularly important for animal and human health. The pathotypes are characterized by virulence factors responsible for different clinical forms. EPEC strains produce intimin, an adherence factor that allows adhesion to enterocytes, inducing microvilli loss and consequent diarrhea [4]. NTEC strains have different virulence factors, including fimbrial and afimbrial adhesins, and toxins; the cytolethal distending toxin (Cdt) and the cytotoxic necrotizing factors CNF1 and CNF2 are involved in necrosis of eukaryotic cells and alteration of the macrophage function [43,44]. STEC strains produce two types of Shiga-toxin (Stx1 and Stx2 toxins) which are toxic to colonic, ileal epithelial, and endothelial cells [45,46]. Strains belonging to these three pathotypes may cause further issues because they can be resistant to one or more antibiotics. Therefore, they can be difficult to treat, but they can also act as sources of antimicrobial resistance genes for other bacteria [1,47].

Nine *E. coli* strains, five NTEC and four EPEC, analyzed in this study were previously isolated from feces collected from seagulls, and the STEC strain from feces of an owl. Even though these strains were susceptible to all, or almost all, tested antibiotics, isolates belonging to these pathotypes can be severe threats for other animals and mainly for humans, who may become infected through direct or indirect contact with dropping of infected birds. Owls are carnivorous birds of prey that live on a diet of insects, earthworms, amphibians, reptiles, birds, mice, rats, shrews, lagomorphs, voles, and moles [48], all potential sources of *E. coli* [49]. Owls are wild birds living far from urban areas, but contact with humans is possible when they are recovered in rescue centers or zoos. Seagulls are migratory birds which cross extensive portions of the coastline areas, contaminating different environments and contributing to the spreading of pathogens, including zoonotic ones such as *E. coli* [50,51,52].

## 5. Conclusions

In our survey, EOs from *O. vulgare*, *S. montana*, and *T. vulgaris* showed a good activity against the analyzed *E. coli* pathogenic strains, which represent a serious risk for the health of animals and humans, and the activity seemed to be increased when a blend containing the three oils in equal parts was used. The use of a mixture could be useful because the employment of more EOs may include antimicrobial activity against more pathogens. The preliminary obtained results suggest that products with these EOs could be used as disinfectants in sprays, foggers, or solution preparations, on surfaces of animal cages, on instruments, and in little areas. In particular, after accurate cleaning to remove dirty material, preparations containing EOs could be employed for a decontaminating action.

However, this preliminary study suggests testing the selected oils with more bacterial strains to confirm their antimicrobial activity; analyses to assess the Eos’ efficacy in the presence of bacterial biofilms should also be performed. In addition, field tests are necessary to verify the Eos’ efficacy in practical use and to compare them with traditional products.

## Figures and Tables

**Table 1:** Results of the MIC determination in relation to the analyzed bacterial strain and the essential oil.

Bacterial Strain	*Origanum* *vulgare* (%)	*Satureja* *montana* (%)	*Thymus* *vulgaris* (%)	Mixture (%)
NTEC 1	0.156	0.078	0.039	≤0.0195
NTEC 2 *	0.078	0.156	≤0.0195	≤0.0195
NTEC 3	0.078	0.078	0.039	≤0.0195
NTEC 4 **	0.078	0.078	0.156	0.039
NTEC 5 **	0.039	0.078	0.156	0.039
EPEC 1	0.078	0.078	≤0.0195	≤0.0195
EPEC 2	0.078	0.039	0.039	≤0.0195
EPEC 3	0.078	0.039	0.039	≤0.0195
EPEC 4	0.039	≤0.0195	0.078	≤0.0195
STEC 1 **	0.156	0.156	0.039	0.039

Legend. *: resistant to trimethoprim-sulfamethoxazole; **: resistant to amoxicillin-clavulanate and ampicillin.

## Data Availability

All data are reported in the manuscript.
